# Simultaneous All-Parameters Calibration and Assessment of a Stereo Camera Pair Using a Scale Bar

**DOI:** 10.3390/s18113964

**Published:** 2018-11-15

**Authors:** Peng Sun, Nai-Guang Lu, Ming-Li Dong, Bi-Xi Yan, Jun Wang

**Affiliations:** 1 Institute of Information Photonics and Optical Communications, Beijing University of Posts and Telecommunications, Beijing 100876, China; sunpeng@bistu.edu.cn (P.S.); nglv2002@163.com (N.-G.L.); 2 Beijing Key Laboratory: Measurement and Control of Mechanical and Electrical System, Beijing Information Science and Technology University, Beijing 100192, China; 3 School of Instrument Science and Opto-Electronics Engineering, Beijing Information Science and Technology University, Beijing 100192, China; yanbx@bistu.edu.cn (B.-X.Y.); wangjun@bistu.edu.cn (J.W.)

**Keywords:** stereo photogrammetry, 3D vision, self-calibration, relative orientation, scale bar

## Abstract

Highly accurate and easy-to-operate calibration (to determine the interior and distortion parameters) and orientation (to determine the exterior parameters) methods for cameras in large volume is a very important topic for expanding the application scope of 3D vision and photogrammetry techniques. This paper proposes a method for simultaneously calibrating, orienting and assessing multi-camera 3D measurement systems in large measurement volume scenarios. The primary idea is building 3D point and length arrays by moving a scale bar in the measurement volume and then conducting a self-calibrating bundle adjustment that involves all the image points and lengths of both cameras. Relative exterior parameters between the camera pair are estimated by the five point relative orientation method. The interior, distortion parameters of each camera and the relative exterior parameters are optimized through bundle adjustment of the network geometry that is strengthened through applying the distance constraints. This method provides both internal precision and external accuracy assessment of the calibration performance. Simulations and real data experiments are designed and conducted to validate the effectivity of the method and analyze its performance under different network geometries. The RMSE of length measurement is less than 0.25 mm and the relative precision is higher than 1/25,000 for a two camera system calibrated by the proposed method in a volume of 12 m × 8 m × 4 m. Compared with the state-of-the-art point array self-calibrating bundle adjustment method, the proposed method is easier to operate and can significantly reduce systematic errors caused by wrong scaling.

## 1. Introduction

Photogrammetry is a technique for measuring spatial geometric quantities through obtaining, measuring and analyzing images of targeted or featured points. Based on different sensor configurations, photogrammetric systems are categorized into offline and online systems [1]. Generally, an offline system uses a single camera to take multiple and sequential images from different positions and orientations. The typical measurement accuracy lies between 1/50,000 and 1/100,000. That substantially results from the 1/20–1/30 pixels target measurement accuracy and the self-calibrating bundle adjustment algorithm [2].

Unlike offline systems, an online system uses two or more cameras to capture photos synchronously and reconstruct space points at any time moment. They are generally applied to movement and deformation inspection during a certain time period. Online systems are reported in industrial applications such as foil material deformation investigation [3], concrete probe crack detection and analysis [4], aircraft wing and rotor blade deformation measurement [5], wind turbine blade rotation measurement and dynamic strain analyses [6,7], concrete beams deflection measurement [8], bridge deformation measurement [9], vibration and acceleration measurement [10,11], membrane roof structure deformation analysis [12], building structure vibration and collapse measurement [13,14], and steel beam deformation measurement [15]. This noncontact and accurate technique is still finding more potential applications.

Online systems can also be applied to medical positioning and navigation [16]. These systems are generally composed of a rigid two camera tracking system, LED or sphere Retro Reflect Targets (RRT) and hand held probes. NDI Polaris (NDI, Waterloo, ON, Canada) and Axios CamBar (AXIOS 3D, Oldenburg, Lower Saxony, Germany) are the leading systems. Generally, the measurement distance is less than 3 m while the coordinate measurement accuracy is about 0.3 mm and length accuracy about 1.0 mm. In order to achieve this, these camera pairs are calibrated and oriented with the help of Coordinate Measuring Machine (CMM) [17] or special calibration frames in laboratory in an independent process before measurement.

Online systems equipped with high frame rate cameras are also employed in 3D motion tracking, such as the Vicon (Vicon, Denver, CO, USA) and Qualisys (Qualisys, Gothenburg, Sweden) systems. In these systems, the number of cameras and their positions vary with the measurement volumes which puts forward the requirement for calibration methods that can be operated on-site. Indeed, these 3D tracking systems are orientated by moving a ‘T’ or ‘L’ shaped object in the measurement volume. Generally speaking, motion tracking systems are featured in real-time 3D data, flexibility and easy operation, rather than high accuracy.

For multi-camera photogrammetric systems, interior, distortion and exterior parameters are crucial for 3D reconstruction and need to be accurately and precisely calibrated before any measurement. There are mainly two methods for calibrating and orienting multi-camera systems for large volume applications: the point array self-calibrating bundle adjustment method and the moving scale bar method. The point array self-calibrating bundle adjustment method is the most accurate and widely used method. Using convergent and rotated images of well distributed stable 3D points, bundle adjustment recovers parameters of the camera and coordinates of the point array. There is no need to have any prior information about the 3D point coordinates. For example, a fixed station triple camera system is calibrated in a rotatory test field composed of points and scale bars [18,19]. However, sometimes in practice, it is difficult to build a well distributed and stable 3D point array, so the moving scale bar method was developed.

Originally, the moving scale bar method was developed for exterior parameter determination. Hadem [20] investigated the calibration precision of stereo and triple camera systems under different geometric configurations. Specifically, he simulated and experimented with camera calibration in a pure length test field, and he mentioned that a moving scale bar can be easily adapted to stereo camera system calibration. Patterson [21,22] determined the relative orientation of a stereo camera light pen system using a moving scale bar. For these attempts, interior orientations and distortion parameters of the cameras are pre-calibrated before measurement. The accuracy is assessed by length measurement performance according to VDI/VDE CMM norm. Additionally, the author mentioned that length measurement of the scale bar provides on-site accuracy verification. Mass [23] investigated the moving scale bar method using a triple camera system and proposed that the method is able to determine interior orientations and is reliable enough to calibrate multi-camera systems. However, it is required that the cameras be fully or partially calibrated in an offline process [24], and the measurement volume be small, for example 1.5 m × 1.7 m × 1.0 m [23].

This paper proposes a method for calibrating and orienting stereo camera systems. The method uses just a scale bar and can be conducted on-site. Unlike previous moving scale bar methods, this method simultaneously obtains interior, distortion and exterior parameters of the cameras without any prior knowledge of the cameras’ characteristics. Additionally, compared with traditional point array bundle adjustment methods, the proposed method does not require construction of 3D point arrays, but achieves comparable accuracy.

The paper is organized as follows: Section 1 introduces the background of this research, including development of online photogrammetry, state-of-the-art techniques for calibrating and orienting online photogrammetric cameras and their limitations. Section 2 elaborates the mathematical models, computational algorithms and the precision and accuracy assessment theory of the proposed method. Section 3 and Section 4 report the simulations and experiments designed to test the method. Advantages of the method, advices for improving calibration performance and potential practical applications are summarized in the conclusions.

## 2. Materials and Methods

A scale bar is an alloy or carbon fiber bar that has two photogrammetric RRTs fixed at each end. The length between the two RRTs is measured or calibrated by instruments with high (several micrometer) accuracy. One of the measurement instruments is composed by an interferometer, a microscope and a granite rail [25,26]. Generally, a scale bar performs in photogrammetry as a metric for true scale, especially in multi-image offline systems. In this paper, the scale bar is used as a calibrating tool for multi-camera online systems.

**Figure 1:**
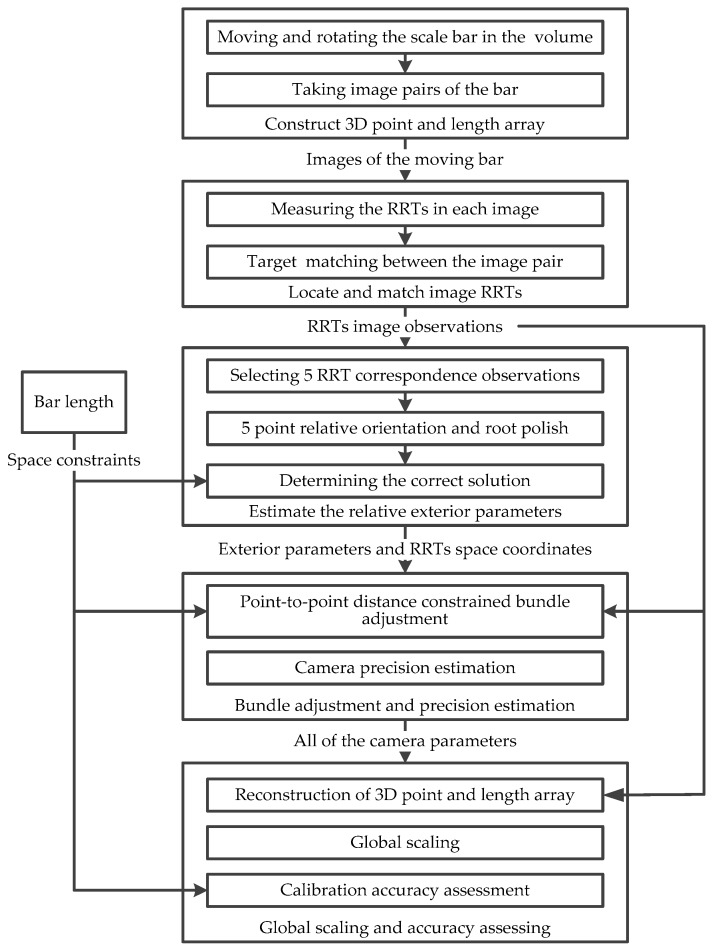
Key components and procedures of the method.

After being settled according to the measurement volume and surroundings, the camera pair is calibrated and oriented following the proposed method outlines in Figure 1.

### 2.1. Construction of 3D Point and Length Array

The bar is moved in the measurement volume to different locations that are uniformly distributed in the measurement volume. At each location, the bar is rotated in different orientations. The position and orientation is called an attitude of the bar. After the moving and rotating process, a virtual 3D point array is built by the RRTs of the bar in each attitude. Meanwhile, because the distance between the two RRTs is the length of the scale bar, a virtual 3D length array is built by the bar length in each attitude. The cameras synchronically capture images of the measurement volume and the bar in each attitude.

### 2.2. Locating and Matching the RRTs in Images

The 2D coordinates of the two RRT in every image are determined by computing the grey value centroid of the pixels in each RRT region. Correspondences of the two RRTs between each image pair is determined by relative position of the points in image. More specifically, the right/left/up/down RRT in one image is matched to the right/left/up/down RRT in the other image. The 2D coordinates of the matched RRTs in all image pairs are used for exterior parameter estimation and all-parameter bundle adjustment.

### 2.3. Estimating the Relative Exterior Parameters

The five-point method [27] is used to estimate the essential matrix between the two cameras. At this stage, we only know a guess of the principle distance. The principle point offset coordinates, distortion parameters of each camera are unknown and set to zeros.

Improperly selected five image point pairs may lead to computing degeneration and thus failure of the method. So to avoid this problem, an algorithm is designed for automatically selecting the most suitable five point pairs, taking into account both maximizing distribution dispersion and avoiding collinearity. The strategy is to find five point pairs that are located near the center and four corners of the two camera images by minimizing the following five functions:

(1) $$\left|x_{l}^{i}\right|+\left|y_{l}^{i}\right|+\left|x_{r}^{i}\right|+\left|y_{r}^{i}\right|,\;-x_{l}^{i}-y_{l}^{i}-x_{r}^{i}-y_{r}^{i},\;x_{l}^{i}-y_{l}^{i}+x_{r}^{i}-y_{r}^{i},\;x_{l}^{i}+y_{l}^{i}+x_{r}^{i}+y_{r}^{i}\;\mathrm{and}\;-x_{l}^{i}+y_{l}^{i}-x_{r}^{i}+y_{r}^{i}\;$$

**Figure 2:**
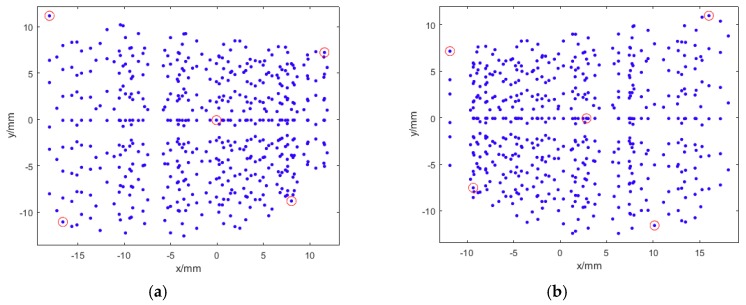
All the 2D RRTs centroids (blue dots) and the selected five correspondences (red circle) for relative orientation. (**a**) RRT points of the left camera; (**b**) RRT points of the right camera.

Figure 2 illustrates all the image RRT points and the selected five point pairs.

The computed essential matrices are globally optimized by the root polish algorithm [28] using all the matched RRTs. After that, the essential matrices are decomposed into the rotation matrices and translation vectors from which we can get the exterior angle and translation parameters. Generally, at least two geometric network structures of the cameras can be obtained and only one is physically correct. In this method, the equalization of the reconstructed lengths in the 3D length array is employed as a spatial constraint to determine the true solution, which is more robust than the widely used image error analysis.

In this part, the relative exterior parameters are inaccurate, the principle distance is just a guess and principle point offset as well as distortions are not dealt with. All of the inaccurate and unknown parameters need further refinement through bundle adjustment to achieve high accuracy and precision.

### 2.4. Self-Calibrating Bundle Adjustment and Precision Estimation

For traditional 3D point array self-calibrating bundle adjustment, large amount of convergent images are essential to handle the severe correlations between unknown parameters and to achieve reliable and precise results, so in theory, calibrating cameras through bundle adjustment using only one image pair of pure 3D point array is impossible, but moving a scale bar gives not only 3D points but also point-to-point distances which, as spatial constraints, greatly strengthen the two-camera network and can be introduced into bundle adjustment to enable self-calibration.

The projecting model of a 3D point into the image pair is expressed by the following implicit collinear equations [29,30]:

(2) $$\begin{array}{c} \underset{\left(2\times 1\right)}{xy_{li}}=f_{}(\begin{array}{ccc} \underset{\left(8\times 1\right)}{I_{l}} & & \underset{\left(3\times 1\right)}{X_{i}} \end{array})\\ \underset{\left(2\times 1\right)}{xy_{ri}}=f_{}(\begin{array}{ccc} \underset{\left(8\times 1\right)}{I_{r}} & \underset{\left(6\times 1\right)}{E_{r}} & \underset{\left(3\times 1\right)}{X_{i}} \end{array}) \end{array}.$$

In Equation (2), the subscripts *l* and *r* mean the left and right camera; *xy* is the image coordinate vector. *I* is the interior parameter vector including the principle distance, the principle point offset, the radial distortion and decentering distortion parameters; *E_r_* is the exterior parameter vector of the right camera relative to the left including three angles and three translations; and *X_i_* is the coordinate vector of a 3D point. The linearized correction equations for an image point observation are:

(3) $$\begin{array}{c} \underset{\left(2\times 1\right)}{v_{li}}+\underset{\left(2\times 1\right)}{l_{li}}=\underset{\left(2\times 8\right)}{A_{li}}\underset{\left(8\times 1\right)}{\delta_{l}}+\underset{\left(2\times 3\right)}{B_{li}}\underset{\left(3\times 1\right)}{{\dot{\delta }}_{i}}\\ \underset{\left(2\times 1\right)}{v_{ri}}+\underset{\left(2\times 1\right)}{l_{ri}}=\underset{\left(2\times 14\right)}{A_{ri}}\underset{\left(14\times 1\right)}{\delta_{r}}+\underset{\left(2\times 3\right)}{B_{ri}}\underset{\left(3\times 1\right)}{{\dot{\delta }}_{i}} \end{array}.$$

**Figure 3:**
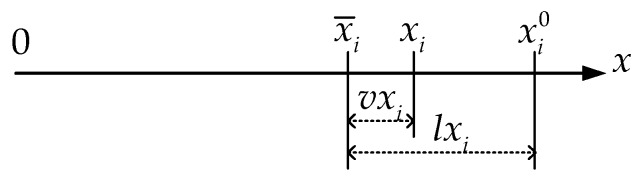
The residual *v* and reduced observation *l* of an image point *i* along *x* axis.

In Equation (3), *v* is the residual vector of an image point that is defined by the disparity vector between the “true” (without error) coordinate $x_{i}$ and the measured image point coordinate ${\bar{x}}_{i}$; *l* is the reduced observation vector that is defined by the disparity vector between the measured image point coordinate ${\bar{x}}_{i}$ and the computed image coordinate $x_{i}^{0}$ using the approximate camera parameters. Figure 3 illustrates the *x* axis component of *v* and *l* of an image point *i*. *A* is the Jaccobian matrix of *f* with respect to camera interior, distortion and exterior parameters; *B* is the Jaccobian matrix of *f* with respect to space coordinates; *δ* and $\dot{\delta }$ are the corrections of the camera parameters and the spatial coordinates, respectively.

*n* scale bars provide 2*n* 3D points and *n* point-to-point distances. Considering the *m*-th bar length:

(4) $$s_{m}=g(\begin{array}{cc} X_{m_{1}} & X_{m_{2}} \end{array})=\sqrt{{\left(X_{m_{1}}-X_{m_{2}}\right)}^{2}+{\left(Y_{m_{1}}-Y_{m_{2}}\right)}^{2}+{\left(Z_{m_{1}}-Z_{m_{2}}\right)}^{2}},$$

where, *m*_1_ and *m*_2_ denote the two endpoints of the bar. Because Equation (4) is nonlinear, they need to be linearized before participating the bundle adjustment. The linearized correction equation for a spatial point-to-point distance constraint is:

(5) $$\underset{\left(1\times 1\right)}{vs_{m}}+\underset{\left(1\times 1\right)}{ls_{m}}=\underset{\left(1\times 3\right)}{C_{m_{1}}}\underset{\left(3\times 1\right)}{{\dot{\delta }}_{m_{1}}}+\underset{\left(1\times 3\right)}{C_{m_{2}}}\underset{\left(3\times 1\right)}{{\dot{\delta }}_{m_{2}}},$$

where, *C* is the Jaccobian matrix of Equation (4) with respect to the coordinates of each endpoint.

Point-to-point distances are incorporated into bundle adjustment to avoid rank defect of the normal equation and also to eliminate correlations between unknown parameters. For a two camera system imaging *n* scale bars, the extended correction equation that involves all the image point observations and point-to-point distance constraints can be written as:

(5) $$v+l=A\bar{\delta }\;\left[\begin{array}{c} v_{1,11}\\ v_{1,12}\\ \vdots \\ v_{1,n1}\\ v_{1,n2}\\ v_{2,11}\\ v_{2,12}\\ \vdots \\ v_{2,n1}\\ v_{2,n2}\\ vs_{1}\\ \vdots \\ vs_{n} \end{array}\right]+\left[\begin{array}{c} l_{1,11}\\ l_{1,12}\\ \vdots \\ l_{1,n1}\\ l_{1,n2}\\ l_{2,11}\\ l_{2,12}\\ \vdots \\ l_{2,n1}\\ l_{2,n2}\\ ls_{1}\\ \vdots \\ ls_{n} \end{array}\right]=\left[\begin{array}{ccccccc} A_{1,11} & 0 & B_{1,11} & 0 & \cdots & 0 & 0\\ A_{1,12} & 0 & 0 & B_{1,12} & \cdots & 0 & 0\\ \vdots & \vdots & \vdots & \vdots & \vdots & \vdots & \vdots \\ A_{1,n1} & 0 & 0 & 0 & \cdots & B_{1,n1} & 0\\ A_{1,n2} & 0 & 0 & 0 & \cdots & 0 & B_{1,n2}\\ 0 & A_{2,11} & B_{2,11} & 0 & \cdots & 0 & 0\\ 0 & A_{2,12} & 0 & B_{2,12} & \cdots & 0 & 0\\ \vdots & \vdots & \vdots & \vdots & \vdots & \vdots & \vdots \\ 0 & A_{2,n1} & 0 & 0 & \cdots & B_{2,n1} & 0\\ 0 & A_{2,n2} & 0 & 0 & \cdots & 0 & B_{2,n2}\\ 0 & 0 & C_{11} & C_{12} & \cdots & 0 & 0\\ \vdots & \vdots & \vdots & \vdots & \vdots & \vdots & \vdots \\ 0 & 0 & 0 & 0 & \cdots & C_{n1} & C_{n2} \end{array}\right]\left[\begin{array}{c} \delta_{1}\\ \delta_{2}\\ {\dot{\delta }}_{11}\\ {\dot{\delta }}_{12}\\ \vdots \\ {\dot{\delta }}_{n1}\\ {\dot{\delta }}_{n2} \end{array}\right],$$

where the subscripts (*i*, *j, k*) denote the *k*-th (*k* = 1, 2) endpoint of the *j*-th (*j* = 1, 2, …, *n*) distance in the *i*-th (*i* = 1, 2) image. The normal equation is:

(7) $$\begin{array}{c} (A^{T}PA)\bar{\delta }=A^{T}Pl\Rightarrow N\bar{\delta }=W\\ \left[\begin{array}{cc} \underset{\left(22\times 22\right)}{N_{11}} & \underset{\left(22\times 6n\right)}{N_{12}}\\ \underset{\left(6n\times 22\right)}{N_{12}^{T}} & \underset{\left(6n\times 6n\right)}{N_{22}} \end{array}\right]\left[\begin{array}{c} \underset{\left(22\times 1\right)}{\delta }\\ \underset{\left(6n\times 1\right)}{\dot{\delta }} \end{array}\right]=\left[\begin{array}{c} \underset{\left(22\times 1\right)}{W_{1}}\\ \underset{\left(6n\times 1\right)}{W_{2}} \end{array}\right] \end{array}.$$

In Equation (7), *P* is a diagonal weight matrix of all the image point coordinate and spatial distance observations. Items in Equation (7) are determined by block computation:

$$N_{11}=\left[\begin{array}{cc} \sum_{j=1}^{n}\sum_{k=1}^{2}A_{1,jk}^{T}P_{p}A_{1,jk}^{} & 0\\ 0 & \sum_{j=1}^{n}\sum_{k=1}^{2}A_{2,jk}^{T}P_{p}A_{2,jk}^{} \end{array}\right],$$

$$N_{22}=\left[\begin{array}{ccccc} \sum_{i=1}^{2}B_{i,11}^{T}P_{p}B_{i,11}+C_{11}^{T}P_{l}C_{11} & C_{11}^{T}P_{l}C_{12} & \cdots & 0 & 0\\ C_{12}^{T}P_{l}C_{11} & \sum_{i=1}^{2}B_{i,12}^{T}P_{p}B_{i,12}+C_{12}^{T}P_{l}C_{12} & \cdots & 0 & 0\\ \vdots & \vdots & \vdots & \vdots & \vdots \\ 0 & 0 & \cdots & \sum_{i=1}^{2}B_{i,n1}^{T}P_{p}B_{i,n1}+C_{n1}^{T}P_{l}C_{n1} & C_{n1}^{T}P_{l}C_{n2}\\ 0 & 0 & \cdots & C_{n2}^{T}P_{l}C_{n1} & \sum_{i=1}^{2}B_{i,n2}^{T}P_{p}B_{i,n2}+C_{n2}^{T}P_{l}C_{n2} \end{array}\right],$$

$$N_{12}=\left[\begin{array}{ccccc} A_{1,11}^{T}P_{p}B_{1,11} & A_{1,12}^{T}P_{p}B_{1,12} & \cdots & A_{1,n1}^{T}P_{p}B_{1,n1} & A_{1,n2}^{T}P_{p}B_{1,n2}\\ A_{2,11}^{T}P_{p}B_{2,11} & A_{2,12}^{T}P_{p}B_{2,12} & \cdots & A_{2,n1}^{T}P_{p}B_{2,n1} & A_{2,n2}^{T}P_{p}B_{2,n2} \end{array}\right],$$

$$w_{1}=\left[\begin{array}{c} \sum_{j=1}^{n}\sum_{k=1}^{2}A_{1,jk}^{T}P_{p}l_{1,jk}^{}\\ \sum_{j=1}^{n}\sum_{k=1}^{2}A_{2,jk}^{T}P_{p}l_{2,jk}^{} \end{array}\right],\;w_{1}=\left[\begin{array}{c} \sum_{i=1}^{2}B_{i,11}^{T}P_{p}l_{i,11}+ls_{1}P_{l}C_{11}\\ \sum_{i=1}^{2}B_{i,12}^{T}P_{p}l_{i,12}+ls_{1}P_{l}C_{12}\\ \vdots \\ \sum_{i=1}^{2}B_{i,n1}^{T}P_{p}l_{i,n1}+ls_{n}P_{l}C_{n1}\\ \sum_{i=1}^{2}B_{i,n2}^{T}P_{p}l_{i,n2}+ls_{n}P_{l}C_{n2} \end{array}\right].$$

Assuming that the a priori standard deviations of the image point observation and the spatial distance observation are *s_p_* and *s_l_*, respectively, and the a priori standard deviation of unit weight is *s*_0_, the weight matrices *P_p_* and *P_l_* are determined by:

(8) $$P_{p}=\left[\begin{array}{cc} \frac{s_{0}^{2}}{s_{p}^{2}} & 0\\ 0 & \frac{s_{0}^{2}}{s_{p}^{2}} \end{array}\right],P_{l}=\frac{s_{0}^{2}}{s_{l}^{2}}.$$

Solving Equation (7), we obtain the corrections for camera parameters and endpoint coordinates:

(9) $$\begin{array}{c} \delta ={\left(N_{11}-N_{12}N_{22}^{-1}N_{21}\right)}^{-1}(W_{1}-N_{12}N_{22}^{-1}W_{2})\\ \dot{\delta }=N_{22}^{-1}(W_{2}-N_{21}\delta ) \end{array}.$$

Again, using the block diagonal character of *N*_22_, *δ* can be computed camera by camera and $\dot{\delta }$ can be computed length by length. The estimated camera parameters and 3D point coordinates are updated by the corrections iteratively until the bundle adjustment converges. The iteration converges and is terminated when the maximum of the absolute coordinate corrections of all the 3D points is smaller than 1 μm.

The proposed algorithm is time efficient because block computations eliminate the need for massive matrix inverse or pseudo inverse computation. In addition, the algorithm is unaffected by invisible observations and allows for gross observation detection in the progress of adjustment iteration. Additionally, this method allows both internal precision and external accuracy assessment of the calibrating results. The internal precision is represented by the variance-covariance matrix of all the adjusted unknowns:

(10) $$\underset{\left((22+6n)\times (22+6n)\right)}{C}={\hat{s}}_{0}^{2}\underset{\left((22+6n)\times (22+6n)\right)}{N^{-1}},$$

where *N* is the normal matrix in Equation (7) and the a posteriori standard deviation of unit weight is determined by:

(11) $${\hat{s}}_{0}=\sqrt{\frac{v^{T}Pv}{8n-(22+6n)+n}},$$

where *n* is the number of point-to-point distances and the size of *v* equals 8*n* for a two camera system.

### 2.5. Global Scaling and Accuracy Assessing of the Calibration Results

After bundle adjustment, 3D endpoints can be triangulated using the parameters. Generally, the adjusted results include systematic errors that are caused by wrong scaling and cannot be eliminated through bundle adjustment. Again, the point-to-point distances are utilized to rescale the results. Assuming that the triangulated bar lengths are:

(12) $$L_{1},L_{2},\ldots L_{n}.$$

The rescaling factor is calculated by:

(13) $$K_{s}=\frac{L}{\bar{L}},$$

where *L* is the nominal length of the scale bar and $\bar{L}$ is the average of the triangulated bar lengths in Equation (12). Then the final camera parameters, 3D coordinates and triangulated bar lengths are:

(14) $$I_{l}^{\prime }=I_{l},\;I_{r}^{\prime }=I_{r},\;E_{r}^{\prime }={\left[\begin{array}{cc} E_{r}{\left(1:3\right)}^{T} & K_{s}E_{r}{\left(4:6\right)}^{T} \end{array}\right]}^{T},X_{i}^{\prime }=K_{s}X_{i}\;\mathrm{and}\;L_{i}^{\prime }=K_{s}L_{i}.$$

Besides internal precision, this method provides on-site 3D evaluation of the calibration accuracy. The triangulated lengths $L_{i}^{\prime }(i=1,2,\ldots n)$ provide large amount of length measurements that are distributed in various positions and orientations in the measurement volume. As a result, an evaluation procedure can be carried out following the guidelines of VDI/VDE 2634 norm. Because all the lengths are physically identical, calibration performance assessment through length measurement error is much easier.

Since the length of the scale bar is calibrated by other instruments, the nominal length *L* has error. Assuming that the true length of the scale bar is *L*_0_, we introduce a factor *K* to describe the disparity between *L* and *L*_0_:

(15) $$L=KL_{0},(K\neq 1),$$

Essentially, Equation (15) describes the calibration error of the scale bar length in another way. The triangulated bar lengths $L_{i}^{\prime }(i=1,2,\ldots n)$ in Equation (14) can be rewritten as:

(16) $$L_{i}^{\prime }=K\frac{L_{0}}{\bar{L}}L_{i},(K\neq 1).$$

The absolute error of $L_{i}^{\prime }$ is:

(17) $$e_{i}^{\prime }=KL_{0}(\frac{L_{i}}{\bar{L}}-1),(K\neq 1).$$

It can be derived that the Average (AVG) and Root Mean Square (RMS) values of the error are:

(18) $$AVG(e_{i}^{\prime })=0,\;\mathrm{and}\;\mathrm{RMS}(e_{i}^{\prime })=\frac{KL_{0}}{\bar{L}}\mathrm{RMSE}(L_{i}),$$

where, RMSE(*L_i_*) is the Root Mean Square Error (RMSE) of the triangulated scale lengths.

Further, we define the relative precision of length measurement by:

(19) $$r(L_{i}^{\prime })=\frac{\mathrm{RMSE}({L^{\prime }}_{i})}{L}=\frac{\mathrm{RMS}({e^{\prime }}_{i})}{L}=\frac{\mathrm{RMSE}(L_{i})}{\bar{L}}.$$

In Equation (19), the relative precision is independent of factor *K* and it keeps unchanged under different *K* value. For example, in a calibration process using a nominal *L* = 1000 mm (*K* = 1) bar, ten of the triangulated bar lengths $L_{i}(i=1,2,\ldots 10)$ are:

[999.997 999.986 1000.041 999.969 999.997 999.991 999.998 999.988 1000.024 1000.017]

whose RMSE is 0.020 mm and the relative precision $r(L_{i})$ is 1/50,068.

If *K* of the instrument is 1.000002 (the absolute calibration error is 0.002 mm), the nominal length is 1000.002 mm. According to Equation (16), the rescaled lengths $L_{i}^{\prime }$ are:

[999.999 999.988 1000.043 999.971 999.999 999.993 1000.000 999.990 1000.026 1000.019]

whose relative precision $r(L_{i}^{\prime })$ is also 1/50,068.

And further, if *K* is amplified to 1.2 (the absolute calibration error is 200 mm, which is not possible in practice), the rescaled lengths $L_{i}^{\prime \prime }$ are:

[1199.996 1199.983 1200.050 1199.963 1199.997 1199.989 1199.998 1199.985 1200.029 1200.021]

whose relative precision $r(L_{i}^{\prime \prime })$ is again 1/50,068.

The above example proves Equation (19). The relative precision of length measurement is invariant under different scale bar nominal lengths (different *K* values in Equation (15)), which makes it a good assessment of the calibrating performance of the camera pair.

Additionally, interior, distortion parameters and the relative rotating angles between the two cameras are not affected by the scale factor *K.* These parameters are calibrated with a uniform accuracy, no matter how large the instrument measurement error is, even if we assign a wrong value to *L*. The two cameras can be calibrated precisely without knowing the true length *L*_0_.

## 3. Simulations and Results

A simulation system is developed to verify the effectiveness and evaluate the performance of the proposed method. The system consists of the generating module of control length array, camera projective imaging module, the self-calibrating bundle adjustment module and the 3D reconstruction module. The generating module simulates scale bars that evenly distribute over the measurement volume. The length, positions, orientations of the bar and the scale of the volume can be modified. The imaging module projects endpoints of the bars into the image pair utilizing assigned interior, distortion and exterior parameters. The bundle adjustment module implements the proposed method and calibrates all the unknown parameters of the camera pair. The reconstruction module triangulates all the endpoints and lengths by forward intersection utilizing the calibrating results.

### 3.1. Point and Length Array Construction and Camera Pair Configurations

The bar is set to be 1 m long. Bar positions are evenly distributed in the volume and is one bar length apart from each other. The scale bar is moved to each position and posed in different orientations. It is worth noticing that, if the bar is moved and rotated in a single plane, self-calibrating of the camera parameters fails. That is because there is a great correlation between the interior parameters (principle distance, principle point coordinates) and the exterior translation parameters, and planar objects do not provide sufficient information to handle the correlation. As a result, after bundle adjustment, these parameter determinations show very large standard deviations, which means that the calibration results are not precise and thus not reliable.

**Figure 4:**
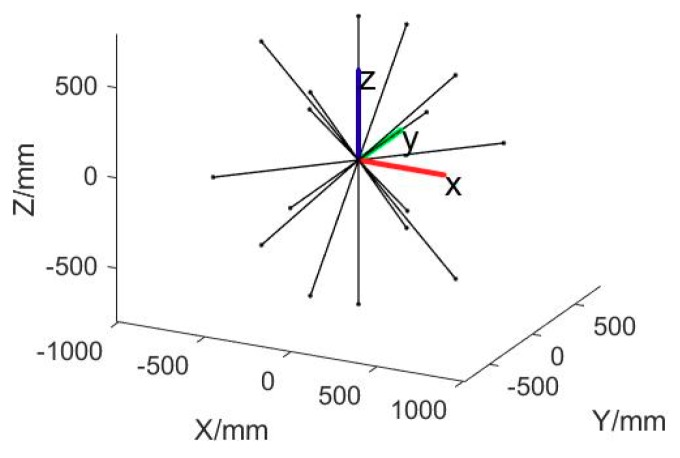
The six orientations of the bar in one position.

**Figure 5:**
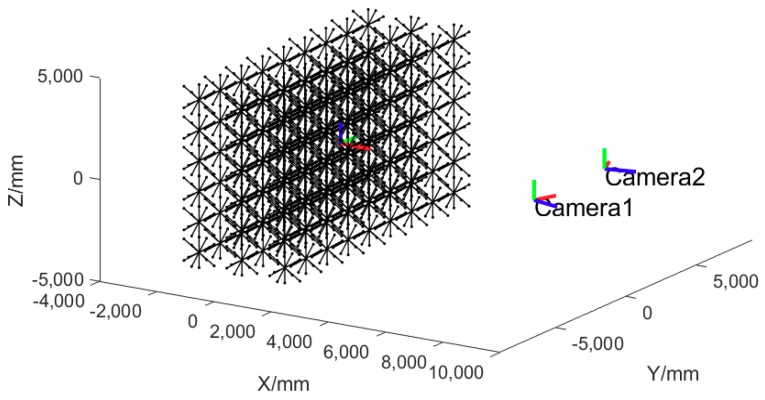
The simulated control length array and the camera pair.

We use multi plane motion and out-of-plane rotations to provide the bundle adjustment process with diverse orientation length constraints and thus optimize the parameters to adapt to different orientations. As a result, uniform 3D measurement accuracy can be achieved in different orientations. Figure 4 shows the six orientations of the bar in one position. Figure 5 demonstrates the simulated point and length array and the camera pair.

In this simulation, the measurement volume is 12 m (length) × 8 m (height) × 4 m (depth). The resolution of the cameras is 4872 × 3248 pixels and the pixel size is 7.4 µm × 7.4 µm. The interior and distortion parameters are set with the values of the real cameras.

The cameras are directed at the center of the measurement volume, and they are 8 m away from the volume and 5 m apart from each other which thus forms a 34.708 degrees intersection angle. Normally distributed image errors of *σ* = 0.2 µm are added to the projected image coordinates of each endpoint. For the cameras simulated, *σ* = 0.2 µm indicates a 1/37 pixels image measurement precision which can be achieved through utilizing RRTs and appropriate image measurement algorithm.

### 3.2. Accuracy and Precision Analysis

In the simulation, only a guess value of 20 mm is assigned to the principle distance. Other interior and distortion parameters are set to zeros. The five point and root polish methods give good estimations of the relative exterior parameters and thus the proposed self-calibrating bundle adjustment converges generally within three iterations.

In the simulation, *s_p_* and *s_l_* are set to 0.0002 mm and 0.2 mm respectively, and *s*_0_ is set to equal *s_p_*. The self-calibrating bundle adjustment refines and optimizes all of the camera parameters and spatial coordinates. A posterior standard deviation of unit weight ${\hat{s}}_{0}$ = 0.00018 mm is obtained, which indicates a good consistency between the a priori and a posterior standard deviation.

The standard deviations of the interior and distortion parameters of the two cameras, the relative exterior parameters and the 3D coordinates of the endpoints can be computed following Equation (10). Table 1 lists the interior, distortion parameter determinations and their standard deviations from the bundle adjustment. The undistortion equation of an image point (*x*, *y*) is:

(20) $$\begin{array}{l} x_{u}=x-x_{0}+\Delta x\\ y_{u}=y-y_{0}+\Delta y \end{array}$$

in which, *x_u_* and *y_u_* are the distortion-free/undistorted coordinates of the point; *x*_0_ and *y*_0_ are the offset coordinates of the principle point in the image; Δ*x* and Δ*y* are the distortions along image *x* and *y* axis respectively. Δ*x* and Δ*y* are calculated by:

(21) $$\begin{array}{l} \Delta x=\bar{x}(K_{1}r^{2}+K_{2}r^{4}+K_{3}r^{6})+P_{1}(2{\bar{x}}^{2}+r^{2})+2P_{2}\bar{x}\bar{y}\\ \Delta y=\bar{y}(K_{1}r^{2}+K_{2}r^{4}+K_{3}r^{6})+P_{2}(2{\bar{y}}^{2}+r^{2})+2P_{1}\bar{x}\bar{y} \end{array}$$

in which, $\bar{x}=x-x_{0},\bar{y}=y-y_{0},r=\sqrt{{\bar{x}}^{2}+{\bar{y}}^{2}}$; *K*_1_, *K*_2_ and *K*_3_ are the radial distortion parameters; *P*_1_ and *P*_2_ are the tangential distortion parameters.

Table 2 lists the relative exterior parameter determinations and their standard deviations from the bundle adjustment, and Table 3 lists the mean standard deviations of the 3D coordinates of all the end points from the bundle adjustment.

From the above results, it can be seen that the bundle adjustment successfully and precisely determines the interior, distortion, and exterior parameters of both cameras as well as the spatial coordinates of the endpoints. Besides internal standard deviations, the reconstructed lengths of the scale bar provide an external evaluation of the calibration accuracy. Table 4 exhibits the results of the triangulated distances versus the known bar length.

**Figure 6:**
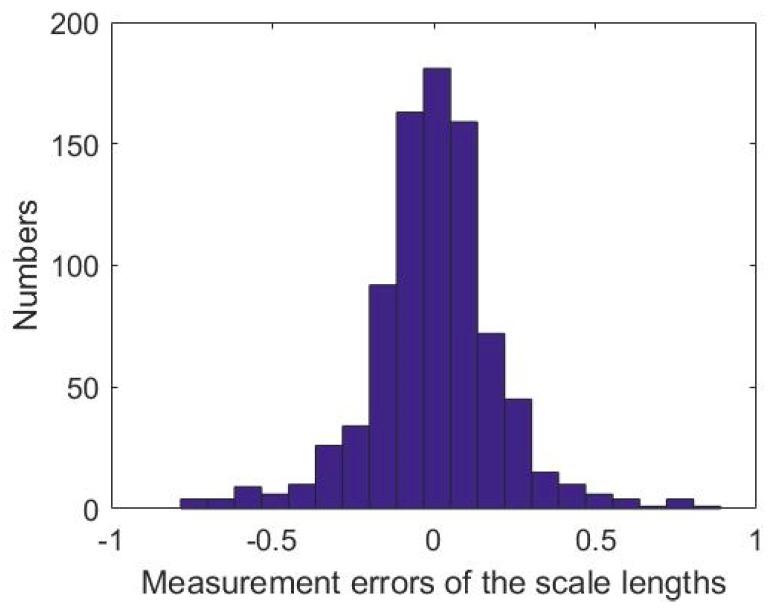
Histogram of the length reconstructing errors.

Figure 6 shows the histogram of the errors of the reconstructed lengths. The errors demonstrate a normal distribution which means that no systematic error components exist and that the functional and the stochastic models of the method are correctly and completely built up.

### 3.3. Performances of the Method under Different Spatial Geometric Configurations

In this part, simulations are carried out to analyze the performance of the proposed method when calibrating camera pairs in different measurement volume scales, using different bar lengths and with different intersection angles.

**Figure 7:**
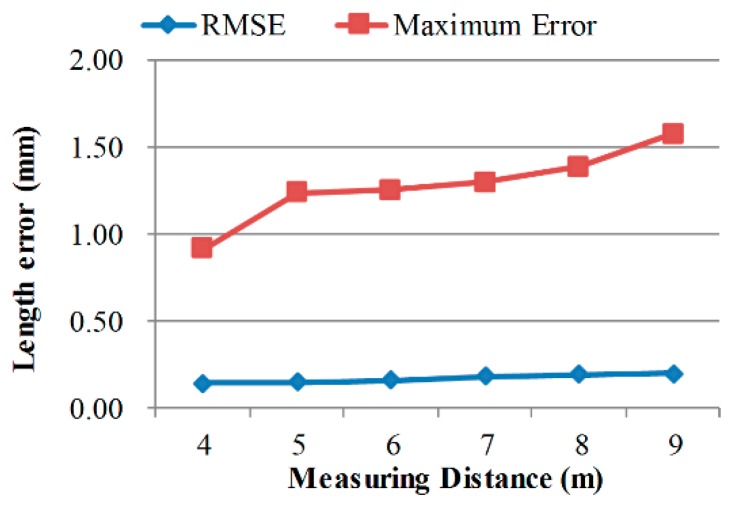
Calibration performances at different measuring distances.

**Figure 8:**
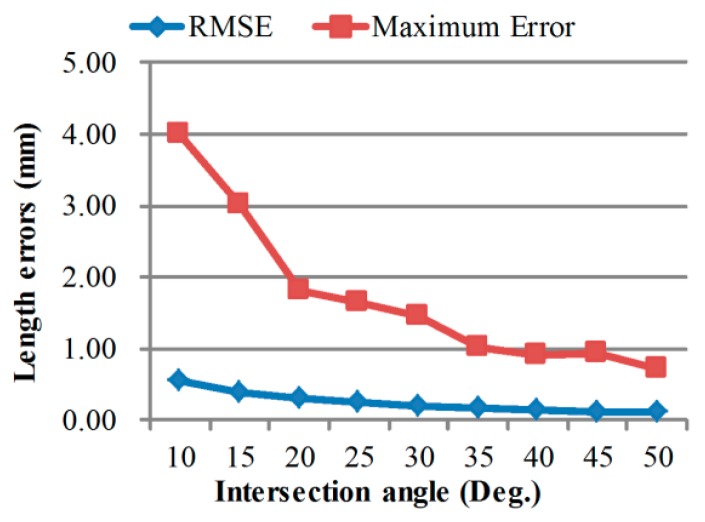
Calibration performances under different camera intersection angles.

**Figure 9:**
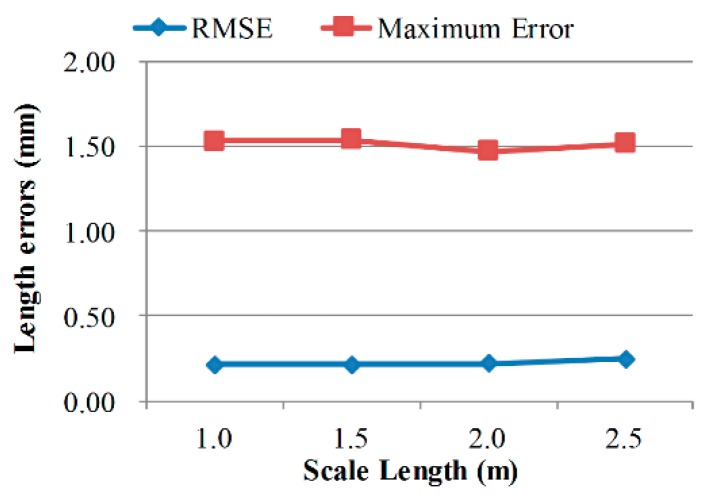
Calibration performances when using different calibrating bar lengths.

For a stereo camera system with a specific intersection angle, the scale of measurement volume is dependent on the measuring distance. Figure 7, Figure 8 and Figure 9 exhibit the calibrating performances, represented by bar length measurement errors, under different geometric configurations.

Calibration accuracy improves in smaller volumes and with larger intersection angles, which is consistent with normal knowledge. What is interesting is that, calibrating accuracy almost keeps unchanged when using scale bars with different lengths. Thus, it can be deduced that, the measuring error of longer distances in the same volume using the calibrated camera pair will be similar to the scale bar length measuring error. Further, the extended relative precision of length measurement in this volume is:

(22) $$r_{e}=\frac{k}{D}\mathrm{RMSE}(L_{i}),$$

where, *k* is a confidence interval integer, *D* is the scale of the measurement volume, RMSE(*L_i_*) is the RMSE of bar length measurement as in Equation (18). For the calibrating results in Table 4, the relative precision is nearly 1/25,000 when *k* equals 3.

### 3.4. Accuracy Comparison with the Point Array Self-Calibrating Bundle Adjustment Method

The point array self-calibration bundle adjustment method is widely used in camera calibration and orientation. This method takes multiple photos of an arbitrary but stable 3D array of points, and then conducts a bundle adjustment to solve the interior, distortion, extrinsic camera parameters and 3D point coordinates. Generally, only one camera is calibrated by the point array bundle adjustment while in our method the two cameras are calibrated simultaneously.

**Figure 10:**
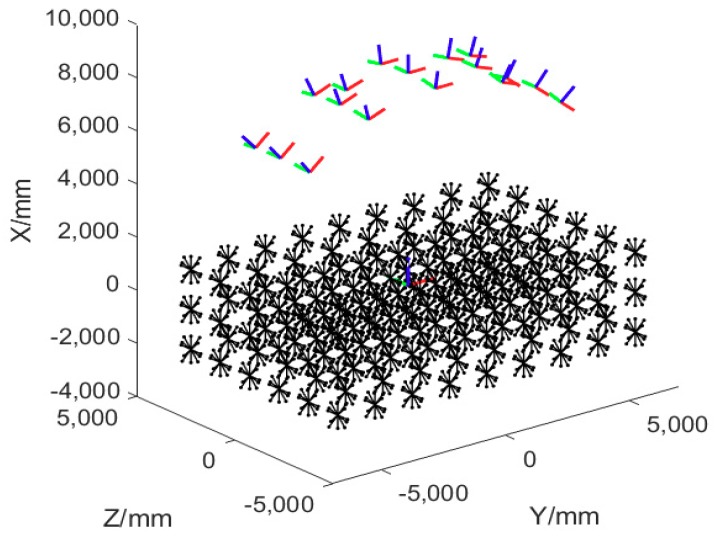
The point array and the camera stations.

The scale bar endpoints in Figure 5 are used to calibrate each camera by the point array self-calibrating bundle adjustment method. For each camera, seventeen convergent pictures of the point array are taken at stations evenly distributed in front of the point array. One of the pictures is taken at the station for stereo measurement and at least one picture is taken orthogonally. Figure 10 demonstrates the camera stations and the point array (the scene is rotated for better visualization of the camera stations).

Image errors of *σ* = 0.2 µm are added to each simulated image point. Then, point array self-calibration bundle adjustment is conducted using these image data to solve the parameters of the two cameras respectively. Besides reconstruction errors of the bar lengths, measurement errors of a 10 m length along the diagonal of the volume are also introduced to make the comparison between these two methods. Table 5 lists the results of 200 simulations of each method. It can be seen that the proposed method is more accurate and precise. The point array bundle adjustment method shows larger systematic errors and Maximum Errors.

## 4. Real Data Experiments and Results

**Figure 11:**
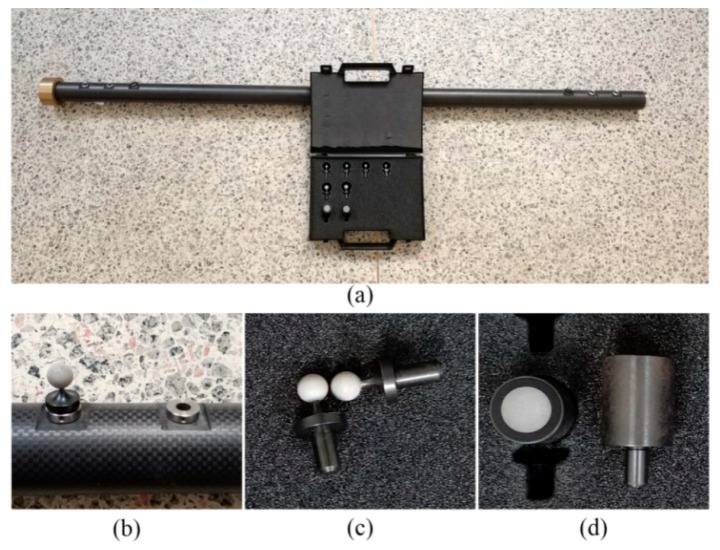
The carbon fiber bar and RRTs. (**a**) The scale bar; (**b**) two of the bushing holes and a target plugged in; (**c**) two spherical targets; (**d**) two planar targets.

Two industrial cameras (GE4900, AVT, Stadtroda, Germany) equipped with two consumer-level lenses (Nikkor 20 mm F/2.8D, Nikon, Tokyo, Japan) are used for real experiments. The resolution of the CCD is 4872 × 3248 pixels and the dimension is 36 mm × 24 mm. Two flashlights (YN 560 III, Yongnuo, Shenzhen, China) are incorporated to provide illumination. A specially designed and manufactured carbon fiber scale bar is employed for calibrating. Figure 11 shows the bar, the spherical and planar RRTs. The bar has symmetrically three bushing holes at each end to brace and fasten the plug-in shafted RRTs. It is convenient to make substitutions for RRTs of different sizes and types. Plugging a pair of RRTs symmetrically in different bushing holes makes three different bar lengths. The lengths are measured on a granite linear rail by a laser interferometer and a microscopic imaging camera. The length measurement accuracy is higher than 2.0 μm.

Three experiments were carried out to validate the proposed method and the simulation results. The cameras are 4 m away from the measurement volume that is 4 m × 3 m × 2 m. The centroid method is employed to measure image RRTs. Target eccentricity is neglected because the computed magnitude according to [31] is less than 0.2 μm across the entire image.

### 4.1. Calibration Performances Using Spherical and Planar Targets

Two types of RRTs are used for camera pair calibration: 9 mm diameter planer circular target and 6 mm diameter spherical target. The length of the bar is set to 0.8 m. Bar positions and orientations in the measurement volume are nearly the same for the two types of target. Table 6 lists the errors. The spherical targets achieve better accuracy because they provide better visibility of the bar from large viewing angle.

### 4.2. Calibration Performances under Different Intersection Angles and Bar Lengths

**Figure 12:**
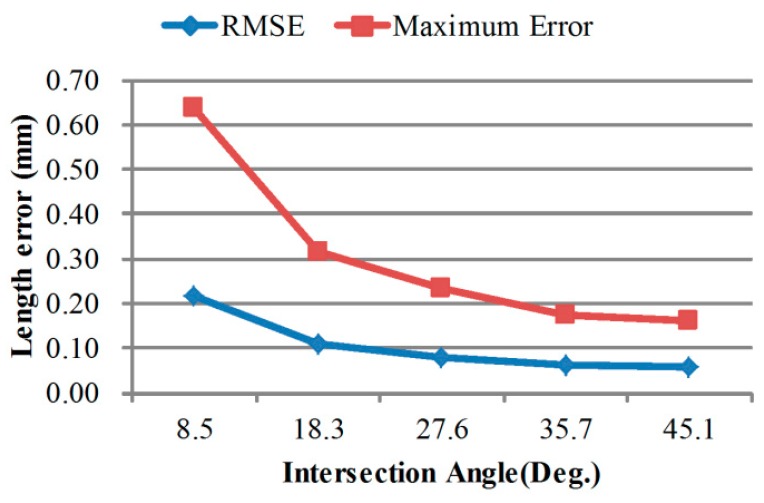
Bar length measurement error varies with intersection angle.

By changing the baseline, we have five different intersection angle configurations and the errors are shown in Figure 12. The plot shows a similar decline tendency as in Figure 8 when intersection angle increases.

The three length configurations of the bar are used respectively for calibrating and the results are listed in Table 7. Almost unchanged RMSE and Maximum Error verify the simulation results in Figure 9.

### 4.3. Comparison with the Ponit Array Bundle Adjustment Method

**Figure 13:**
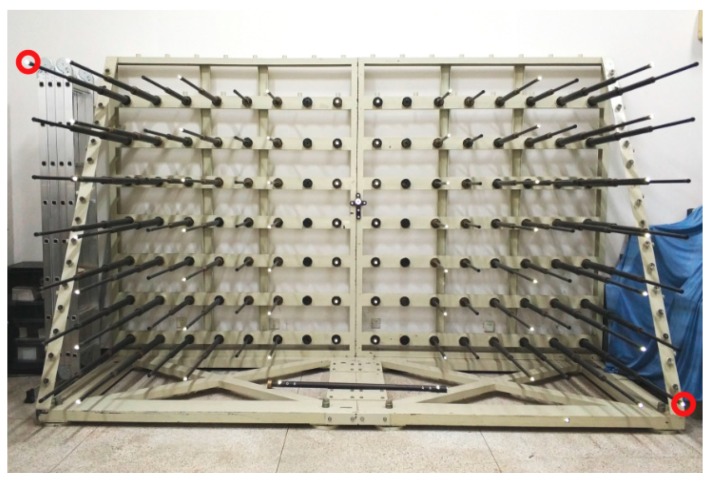
The 3D object used for the comparison experiment.

The comparison experiment is carried out to measure an object that is specially designed for photogrammetric tests. The object is shown in Figure 13.

**Figure 14:**
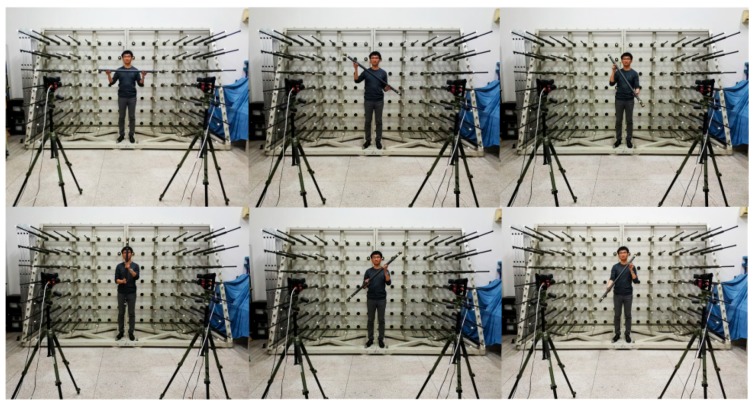
Rotating the bar in one position.

The size of the object is 3.5 m × 2.3 m × 1.5 m. The object contains 58 RRTs (the bright dots) in which 56 are used for point array calibration. The other two RRTs locate in the left-up and right-down corner (indicated by red circles) of the object and are measured by a Laser Tracker (API, LTS 1100, Jessup, MD, USA) and measurement adaptors. The distance between them is called the test length and is used for 3D measurement assessing of the calibration results just like the 10 m diagonal length in simulation. The test length is 4139.810 mm. The cameras are set with the same intersection angle as in the simulations and are calibrated by the proposed method using the bar with planar RRTs and 1078.405 mm length configuration. All the bar lengths and the test length are triangulated using the calibration results. Figure 14 demonstrates rotation of the bar in six orientations.

**Figure 15:**
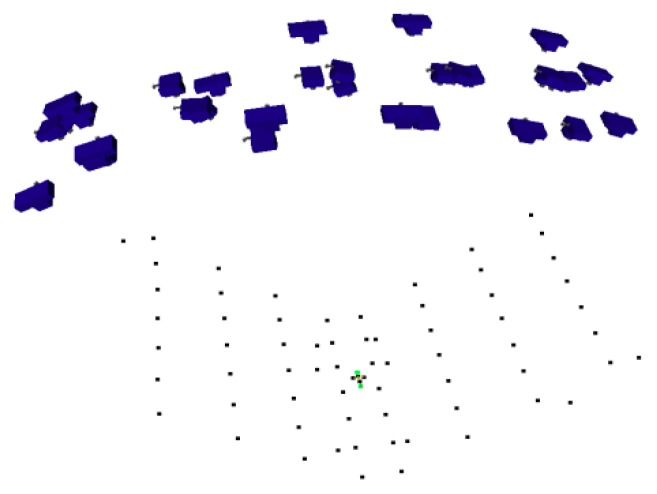
The point array and the photo-taking stations.

Each camera is also calibrated by the point array method with a similar photo-taking style as in Figure 10. Then the bar is moved to construct a length array while the system measures the bar lengths and the test length. Figure 15 shows the network of the point array and the camera stations.

The Mean Error, RMSE and Maximum Error of bar length and test length measurement results in 10 different experiments are listed in Table 8. A very similar comparison result is achieved as Table 5. The proposed method gives better spatial length measurement results.

## 5. Conclusions

This paper proposes a method for simultaneously calibrating and orienting stereo cameras of 3D vision systems in large measurement volume scenarios. A scale bar is moved in the measurement volume to build a 3D point and length array. After imaging the 3D array, the two cameras are calibrated through self-calibration bundle adjustment that is constrained by point-to-point distances. External accuracy can be obtained on-site through analyzing bar length reconstruction errors. Simulations validate effectiveness of the method regarding to the self-calibrating of interior, distortion and exterior camera parameters and meanwhile test its accuracy and precision performance. Moreover, simulations and experiments are carried out to test the influence of the scale bar length, measurement volume, target type and intersection angle on calibration performance. The proposed method does not require stable 3D point array in the measurement volume, and its accuracy will not be affected by the scale bar length. Furthermore, cameras can be accurately calibrated without knowing the true length of the bar. The method achieves better accuracy over the state-of-the-art point array self-calibration bundle adjustment method.

In order to accurately calibrate the interior and distortion parameters, plenty of well/evenly distributed image points are needed, so the bar needs to be moved uniformly in as many positions as possible within the measurement volume. In order to handle the correlation between interior and exterior parameters in bundle adjustment, and thus to guarantee the reliability of the calibration results, the bar needs to be moved in a 3D manner, such as in multi planes and with out-of-plane rotation. Additionally, to achieve uniform triangulation accuracy in different orientations, the bar needs to be rotated uniformly in diverse orientations.

This method can be easily conducted in medium scale volumes within human arm reach, and can be extended to large scale measurement applications with the help of UAVs to carry and operate the scale bar. It can also be used in calibrating small or even micro scale stereo vision systems such as structured light scanner. Compared with planer calibration patterns, scale bars are easier to calibrate, less restricted by camera viewing angle, and has higher image measurement accuracy which will improve calibration accuracy and convenience. Our future works include studies of a rigorous relationship between the motion of the bar and the measurement volume, the relationship between calibrating performance and the number as well as distribution of bar motion positions in the volume, and application of this method in practice.

## Figures and Tables

**Table 1:** Self-calibrating bundle adjustment results of the camera parameters.

Interior Parameters	Left Camera		Right Camera	
	Calibration Results	Standard Deviations	Calibration Results	Standard Deviations
*c* (mm)	20.325	1.542 × 10^−3^	20.320	1.493 × 10^−3^
*x*_0_ (mm)	−0.105	2.245 × 10^−3^	−0.135	2.409 × 10^−3^
*y*_0_ (mm)	0.168	7.423 × 10^−4^	0.247	7.857 × 10^−4^
*K*_1_ (mm^2^)	2.788 × 10^−4^	2.727 × 10^−7^	2.795 × 10^−4^	2.859 × 10^−7^
*K*_2_ (mm^4^)	−4.866 × 10^−7^	1.372 × 10^−9^	−5.034 × 10^−7^	1.575 × 10^−9^
*K*_3_ (mm^6^)	−1.657 × 10^−11^	2.209 × 10^−12^	1.404 × 10^−11^	2.721 × 10^−12^
*P*_1_ (mm)	−7.030 × 10^−6^	1.551 × 10^−6^	−3.102 × 10^−6^	1.620 × 10^−6^
*P*_2_ (mm)	−8.630 × 10^−6^	4.916 × 10^−7^	−8.606 × 10^−6^	5.267 × 10^−7^

**Table 2:** Bundle adjustment results of the relative exterior parameters.

Relative Exterior Parameters	Bundle Adjustment Determinations	Standard Deviations
*Tx* (mm)	4772.757	0.325
*Ty* (mm)	−0.023	0.068
*Tz* (mm)	−1491.147	0.410
*φ* (Deg.)	−34.710	2.845 × 10^−5^
*ω* (Deg.)	0.915 × 10^−3^	8.871 × 10^−5^
*κ* (Deg.)	−1.300 × 10^−3^	2.622 × 10^−5^

**Table 3:** Mean standard deviations of the 3D point coordinate determinations.

Axes	Mean Standard Deviations
*X* (mm)	0.094
*Y* (mm)	0.076
*Z* (mm)	0.203

**Table 4:** Mean Error, RMSE and Maximum Error of the reconstructed distances.

Mean Error (mm)	RMSE (mm)	Maximum Error (mm)
0.005	0.204	1.157

**Table 5:** Length measurement errors of the two methods.

Two Bundle Adjustment Methods	Scale Bar Length Measurement Error (mm)			Spatial Length Measurement Error (mm)		
	Mean Error	RMSE	Maximum Error	Mean Error	RMSE	Maximum Error
Length array	0.008	0.204	1.186	0.101	0.215	0.710
Point array	0.043	0.295	1.664	0.243	0.205	0.820

**Table 6:** Calibration accuracy under different target types.

Target Types	Bar Length Measurement Errors (mm)		RMS of Image Residuals (µm)	
	RMSE	Maximum Error	*x* Axis	*y* Axis
Planar Target	0.192	0.520	0.21	0.33
Sphere Target	0.106	0.296	0.12	0.19

**Table 7:** Bar length measurement errors under different scale bar lengths.

Bar Lengths (mm)	RMSE (mm)	Maximum Error (mm)
880.055	0.123	0.331
970.421	0.132	0.354
1078.405	0.136	0.368

**Table 8:** Length measurement errors of the two methods.

Methods	Scale Bar Length (mm)			Test Length (mm)		
	Mean Error	RMSE	Maximum Error	Mean Error	RMSE	Maximum Error
Length array	0.005	0.150	0.352	0.038	0.151	0.227
Point array	0.031	0.152	0.507	0.134	0.179	0.312
